# Expression Levels of *pvcrt-o* and *pvmdr-1* Are Associated with Chloroquine Resistance and Severe *Plasmodium vivax* Malaria in Patients of the Brazilian Amazon

**DOI:** 10.1371/journal.pone.0105922

**Published:** 2014-08-26

**Authors:** Gisely C. Melo, Wuelton M. Monteiro, André M. Siqueira, Siuhelem R. Silva, Belisa M. L. Magalhães, Aline C. C. Alencar, Andrea Kuehn, Hernando A. del. Portillo, Carmen Fernandez-Becerra, Marcus V. G. Lacerda

**Affiliations:** 1 Universidade do Estado do Amazonas, Manaus, Amazonas, Brazil; 2 Fundação de Medicina Tropical Dr. Heitor Vieira Dourado, Manaus, Amazonas, Brazil; 3 Universidade Paulista UNIP, Manaus, Amazonas, Brazil; 4 Barcelona Centre for International Health Research (CRESIB, Hospital Clínic-Universitat de Barcelona), Barcelona, Spain; 5 Institució Catalana de Recerca i Estudis Avançats (ICREA), Barcelona, Spain; Ehime University, Japan

## Abstract

Molecular markers associated with the increase of chloroquine resistance and disease severity in *Plasmodium vivax* are needed. The objective of this study was to evaluate the expression levels of *pvcrt-o* and *pvmdr-1* genes in a group of patients presenting CQRPv and patients who developed severe complications triggered exclusively by *P. vivax* infection. Two different sets of patients were included to this comprehensive study performed in the Brazilian Amazon: 1) patients with clinically characterized chloroquine-resistant *P. vivax* compared with patients with susceptible parasites from *in* *vivo* studies and 2) patients with severe vivax malaria compared with patients without severity. Quantitative real-time PCR was performed to compare the transcript levels of two main transporters genes, *P. vivax* chloroquine resistance transporter (*pvcrt-o*) and the *P. vivax* multidrug resistance transporter (*pvmdr-1*). Twelve chloroquine resistant cases and other 15 isolates from susceptible cases were included in the first set of patients. For the second set, seven patients with *P. vivax*-attributed severe and 10 mild manifestations were included. Parasites from patients with chloroquine resistance presented up to 6.1 (95% CI: 3.8–14.3) and 2.4 (95% CI: 0.53–9.1) fold increase in *pvcrt-o* and *pvmdr-1* expression levels, respectively, compared to the susceptible group. Parasites from the severe vivax group had a 2.9 (95% CI: 1.1–8.3) and 4.9 (95% CI: 2.3–18.8) fold increase in *pvcrt-o* and *pvmdr-1* expression levels as compared to the control group with mild disease. These findings suggest that chloroquine resistance and clinical severity in *P. vivax* infections are strongly associated with increased expression levels of the *pvcrt-o* and *pvmdr-1* genes likely involved in chloroquine resistance.

## Introduction

Mechanisms underlying severe malaria triggered by *Plasmodium vivax* have been poorly appreciated, mainly the likely higher and more-severe disease burden imposed by increasingly chloroquine-resistant *P. vivax* parasites (CQRPv) [1], [2]. Most common manifestations of severe vivax malaria include severe anemia and respiratory distress, being particularly associated to young age [2]–[4]. Of interest, the majority of reports on severe vivax malaria come from regions where drug-resistant *P. vivax* parasites significantly thread the radical cure and control of this infection [5]. The first reports of CQRPv were made in 1989 [6], [7] from Papua New Guinea and Indonesia. In Brazil, the first case of properly ascertained *in* *vivo* CQRPv was from a patient treated in Manaus, in the Brazilian Amazon [8]. In this city, a subsequent trial assessed the efficacy of standard supervised CQ monotherapy and the proportion of failures was 10.1% [9]. In parallel to the emergence of CQRPv in the Brazilian Amazon, reports of clinical severity exclusively associated with *P. vivax* infection increased substantially [3], [10]–[12].

Based on observational studies, an association between CQRPv and disease severity in *P. vivax* was proposed in the Indonesian Papua [2], although this has not been properly and systematically evaluated. CQR in *P. falciparum* is associated with mutations in the *pfcrt-o* and *pfmdr-1* genes [13], [14] but similar studies with the orthologous genes of *P. vivax*, *pvcrt-o* and *pvmdr-1*, failed to demonstrate a similar association [15]–[17]. Interestingly, a pioneer case of severe disease exclusively associated with *P. vivax,* demonstrated a significant increase on expression levels, not mutations, of these genes [18]. Altogether, these studies suggest the possibility of a significant association of CQRPv and disease severity and that expression levels of *pvcrt-o* and *pvmdr-1* should be better explored as molecular markers of both phenomena to further enhance the understanding of the clinico-epidemiological. The aim of this study was to evaluate the expression levels of *pvcrt-o* and *pvmdr-1* genes in a well-characterized group of patients presenting CQRPv and in a group of patients who developed severe complications triggered by *P. vivax* infection.

## Methods

### Ethics statement

The study was approved by the Ethics Review Board of the *Fundação de Medicina Tropical Heitor Vieira Dourado* (FMT-HVD) (approval number 343/2009). Participants were instructed about the objectives of the study and signed an informed consent. In the case of under 18 years, the consent form was signed by the guardians. Patients diagnosed with malaria were treated according to the guidelines of the Brazilian Ministry of Health [19].

### Study area

The study was carried out from June 2011 to December 2012 in the FMT-HVD, an infectious disease referral center located in Manaus, Western Brazilian Amazon.

### Selection of patients for the CQRPv study

The study included patients with *P. vivax* malaria of both sexes, aged 6 months-60 years, bodyweight greater than 5 kg, presenting blood parasite density from 250 to 100,000 parasites/ml and axillary temperature ≥37.5°C or history of fever in the last 48 hours. Exclusion criteria were: use of antimalarials in the previous 30 days, refusal to be followed up for 42 days and any clinical complication [19]. Patients received supervised treatment with 25 mg/kg of chloroquine (CQ) phosphate over a 3-day period (10 mg/kg on days 0 and 7.5 mg/kg on days 1 and 2). Primaquine was prescribed at the end of follow-up or if disease recurred for 7-day period in the dosage of 0.5 mg/kg per day. Patients who vomited the first dose within 30 minutes after drug ingestion were re-treated with the same dose. Patients were evaluated on days 0, 1, 2, 3, 7, 14, 28 and 42 and, if they felt ill, at any time during the follow-up period. In all sample, blood smear, full blood counts and PCR were performed. On admission (D0) and in the day of recrudescence (DR), 20 ml of intravenous whole blood was also collected for DNA and RNA storage. CQ and desethylchloroquine (DCQ) plasmatic levels were determined only in case of parasitological failure [20]. Three aliquots of 100 µl of whole blood from DR samples were spotted onto filter paper for later analysis by high performance liquid chromatography (HPLC) to estimate the levels of CQ and DCQ as previously described [21], [22]. CQRPv was defined if: 1) recurrences of the parasite with plasma concentrations of CQ higher than 100 ng/dl, or 2) recurrences with smaller concentrations but presenting DR and D0 with the same alleles. The control group consisted of patients with no parasitemia recurrence during the same follow-up period.

### Selection of patients with severe *P. vivax* malaria and patients with mild symptoms

During the same period, admitted patients classified as severe if presenting any of the WHO severity criteria [23], and patients with mild manifestations from the outpatient clinics. On admission, 20 ml of intravenous whole blood was collected for complete blood count, blood biochemistry and for DNA and RNA extraction. Other tests were requested at physician’s discretion. To exclude patients with infection other than *Plasmodium*, all patients also had a blood culture performed for aerobic bacteria and serological tests for leptospirosis (IgM), HIV-1/HIV-2, hepatitis A (anti-HAV IgM), hepatitis B (HBsAg), hepatitis C (anti-HCV), hepatitis D (total anti-HDV) and dengue (RT-PCR). Patients presenting any sign of severity were treated with intravenous artesunate, as recommended by the WHO guidelines [19].

### *P. vivax* malaria diagnosis

Thick blood smear was prepared as recommended by the Walker technique [24] and evaluated by an experienced microscopist [25]. Parasite densities (parasites/µl) were calculated by counting the number of parasites per 500 leukocytes in high magnification fields, and the number of leukocytes/µl per patient. In addition, differential counting of asexual forms (ring-stage parasites, mature trophozoites and schizonts) was made to ensure that there was no difference between groups of cases and controls.

Afterwards, real-time PCR was performed to confirm *P. vivax* mono-infection. The extraction of total DNA from whole blood was performed using the QIAamp DNA Blood Mini Kit (Qiagen, USA), according to the manufacturer’s protocol. The DNA was amplified in an Applied Biosystems 7500 Fast System using primers and TaqMan fluorescence labeled probes for real time PCR [26].

### RNA extraction and expression of *pvcrt-o* and *pvmdr-1* genes

For RNA extraction, 5 ml of whole blood were collected and processed using saponin. Total RNA was purified using Trizol reagent (Invitrogen) according to the manufacturer’s instructions. Furthermore, 2% agarose gel electrophoresis was made to guarantee RNA quality. Amplification reactions were performed using Power SYBR Green PCR Master Mix (Applied Biosystems) and 45 ng of template cDNA prepared from each sample. PCR products were amplified and detected on a 7500 FAST (Applied Biosystems). The primers were

*pvmdr-1*F (AAGGATCAAAGGCAACCCA),

*pvmdr-1*R (TCAGGTTGTTACTGCTGTTGCTATT),

*pvcrt-o*F (ATGTCCAAGATGTGCGACGAT),

*pvcrt-o*R (CTGGTCCCTGTATGCAACTGAC),

*pvtubulin*F (CCAAGAATATGATGTGTGCAAGTG),

*pvtubulin*R (GGCGCAGGCGGTTAGG) [18]. Cycling parameters for PCR were an initial denaturation step at 95°C for 10 minutes, followed by 40 cycles of 95°C for 15 seconds, and 60°C for 1 minute. Samples were run in triplicate. To analyze the relative transcript levels, the threshold cycle value (Ct) of each sample was used to calculate and compare the ΔCt of each sample to that of the *P. vivax* housekeeping gene Sal I β-tubulin; the ΔΔCt was also calculated as *N-fold = 2* ^−ΔΔCT^ [27] to compare the transcript levels of *pvcrt-o* and *pvmdr-1* in the different groups of patients included in this study versus CQ-susceptible patients. After the PCR reaction in real time, an additional step of decoupling the amplicon to generate a melting curve was performed. The presence of only one peak in the dissociation curves reveals the absence of unspecific bands, confirming the specificity of the amplification primers in real time PCR.

Reproducibility of the method (accuracy inter-runs) was assessed through measures of Cts obtained by cDNA quantification of one isolate analyzed 10 times in two runs that were performed on different days, using the same equipment. The results were expressed as mean, standard deviation (SD) and coefficient of variation (CV%). The repeatability of the method (accuracy intra-run) was evaluated by measures of Cts obtained by cDNA quantification of one isolate measured 10 times in the same run. The results were expressed by mean, standard deviation (SD) and coefficient of variation (CV%).

### Sequencing analysis of *pvcrt-o* and *pvmdr-1* genes

All samples from patients presenting CQRPv or patients with severe symptoms and the respective control groups, obtained prior to treatment (on D0), were sequenced for searching mutations suspected to be associated to CQR. Extraction of whole DNA was carried out using a QIAamp DNA Mini Kit (QIAGEN, Germany) according to the manufacturer’s protocol. PCR primers and different reaction conditions used to amplify *pvcrt-o* and *pvmdr-1* gene sequences were made as previously described (Table S1). The amplification products were quantified by NanoDrop 2000 (Thermo Scientific) and sent for sequencing to Macrogen (South Korea). Amino acid sequences were compared with sequences of Sal I (Salvador I): GenBank accession nos. ADE74979.1 for *pvcrt-o* [17] and EU333979.1 for *pvmdr-1* [28]. The deduced amino acid sequences were aligned and analyzed using Mutation Surveyor. The *pvmdr-1* and *pvcrt-o* gene sequences were deposited in GenBank under the accession numbers KM016489 to KM016517 and KM016518 to KM016532, respectively.

### Polymorphism analysis of *msp1*F3 and MS2 molecular markers for patients with CQRPv

PCRs were performed in 20 µl reactions containing 10 µM of each primer, 2 µl of 10×buffer B, 2.5 mM each dNTP, 25 mM MgCl2, 1.5 U Taq DNA polymerase and 5 µl genomic DNA (Table S2). 3 µl of diluted primary PCR product was used as template for the nested PCR (MS2 and *msp1*F3). These polymorphism markers were chosen based on preliminary data from other patients from Manaus (unpublished data). PCRs were performed in a thermocycler with conditions as follows: initial denaturation at 95°C for 1 min, followed by 29 cycles (primary PCR) or 24 cycles (nested PCR) of denaturation at 95°C for 30 seconds, annealing at 59°C for 45 sec, elongation at 72°C for 1 min, followed by a final elongation at 72°C for 5 min. PCR products were stored in the dark (plates wrapped in foil) at 4°C until be sent to Macrogen for capillary electrophoresis-based sequencing and analyzed with GeneMarker version 2.6.0.

### Statistical Analyses

Data were analyzed using SPSS version 16.0 for Windows (SPSS Inc Chicago, IL, USA). Normal distribution of data of hemoglobin and gene expression was evaluated with the Kolmogorov-Smirnov test. Linear regression was performed to correlate this variable with hemoglobin and gene expression among hospitalized patients. Spearman’s correlation test was performed to correlate CQ concentration with gene expression in the DR and gene expression at D0 and DR with concentration of RNA. The Wilcoxon rank pairs test was performed to compare CQR patients had paired expression levels at D0 and DR. Chi-square or Fisher’s test was used to test differences in proportions of polymorphism and distribution of asexual blood stages. The genetic variation for each microsatellite locus was measured by calculating the expected heterozygosity (H_E_). H_E_ was calculated using D0 and DR for each locus as  =  [n/(n−1)] [1−∑p_i_^2^] where n is the number of isolates sampled and p_i_ is the frequency of allele i.

## Results

### Reproducibility, repeatability of measurements of Cts and specificity

For reproducibility, amplification of *pvcrt-o* and *pvmdr-1* genes showed average Ct values ranging from 35.14 to 37.93 (mean Ct value 36.82) and from 34.99 to 36.19 (mean Ct value 35.44), respectively. Among the twenty measured values, doing in to different runs, the maximum coefficient of variation was 2.34% and 1.12%, for *pvcrt-o* and *pvmdr-1* respectively. For the repeatability, the coefficient of variation was 1.35% for the *pvcrt-o* and 0.75% for *pvmdr-1* in the first run, and 2.77% for the *pvcrt-o* and 1% for *pvmdr-1* in the second run (Table S3).

After the relative quantification, only one peak was observed in the dissociation curves, confirming a good specificity of the primers in real time PCR.

### Gene expression levels from CQR parasites

In this period, in a parallel study to evaluate efficacy of CQ with the 42-day follow-up, with supervised drug administration, 135 patients were enrolled, and 16 were CQR (11.8%). Of these, 12 with adequate RNA storage were included in the analysis. Clinical and laboratorial characteristics of the 12 patients with CQRPv included in this study are presented in Table 1. Out of this total, 83.3% patients were male, with a mean age of 29.0 years. The mean hemoglobin was 13.1 g/dl at D0 and 12.7 g/dl in DR. The geometric mean parasitemia was 1781.7 parasites/µl at D0 and 4206.5 parasites/µl at DR. It was found that mean blood levels of CQ plus DCQ were greater than 100 ng/ml on the DR in 10 of 12 (83.3%) patients with recurring infection. Five patients with CQ-susceptible parasites and mild malaria were selected to generate the relative expression of 12 CQRPv and 15 CQ-susceptible isolates.

**Table 1:** Demographic and clinical characteristics of the study participants admitted to a tertiary health center, Manaus, Amazonas, Brazil.

Code	Sex/Age	DR	Haemoglobinlevel inD0 (g/dL)	Haemoglobinlevel inDR (g/dL)	Chloroquineconcentrations(ng/dL)	Asexualmalarial parasites/µlin D0	Asexualmalarial parasites/µlin DR	Day of RNAIsolation	Genotyping
R1	M/55	27	14	15.1	249	690.0	5695.0	D0/DR	Same
R2	F/9	32	10.2	11.6	61	4347.0	6871.0	D0/DR	Same
R3	M/23	34	13.4	12.6	68	297.0	3734.6	D0/DR	Same
R4	M/42	36	14.2	13.5	256	686.0	436.8	D0/DR	Same
R5	M/13	38	12.4	12.0	103	2582.1	2613.2	D0/DR	Same
R6	M/25	25	15.2	15.6	533	1974.7	5385.6	D0/DR	Same
R7	M/37	28	14.0	14.4	263	2548.0	50.0	D0/DR	Same
R8	M/26	32	13.5	12.9	188	78.0	11096.3	D0	Same
R9	M/9	31	9.4	10.0	230	1136.8	4968.0	D0	Same
R10	M/47	31	12.9	12.0	130	5059.2	4160.0	D0	Different
R11	M/36	34	14.7	14.5	118	2105.6	2853.6	DR	Different
R12	F/44	34	13.6	13.1	111	676.8	2613.2	DR	Same

Day admission (D0). Day of recrudescence (DR).

**Figure 1:**
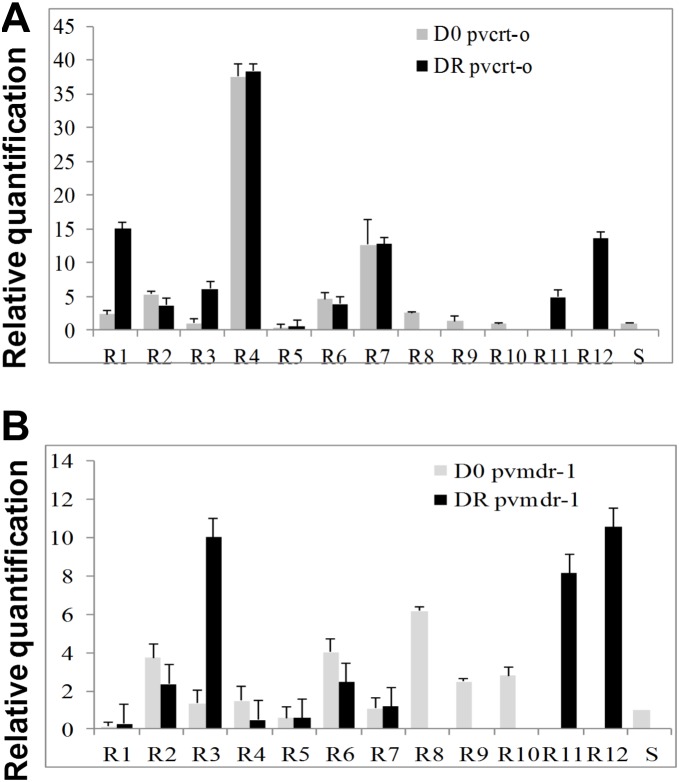
Expression levels of chloroquine resistance genes in patients with CQRPv parasites. Relative quantification of *pvcrt-o* (A) and *pvmdr1* (B) transcripts in total RNA obtained from parasites from patients with chloroquine-resistant *P. vivax* vs a pool of total RNA obtained from 5 patients with CQ-susceptible parasites. Chloroquine-resistant *P. vivax* parasites (R). Chloroquine-susceptible *P. vivax* parasites (S). Day of admission (D0). Day of recrudescence (DR). The error bars in Figures 1 and 3 reflect the average standard error of the Ct.

**Figure 2:**
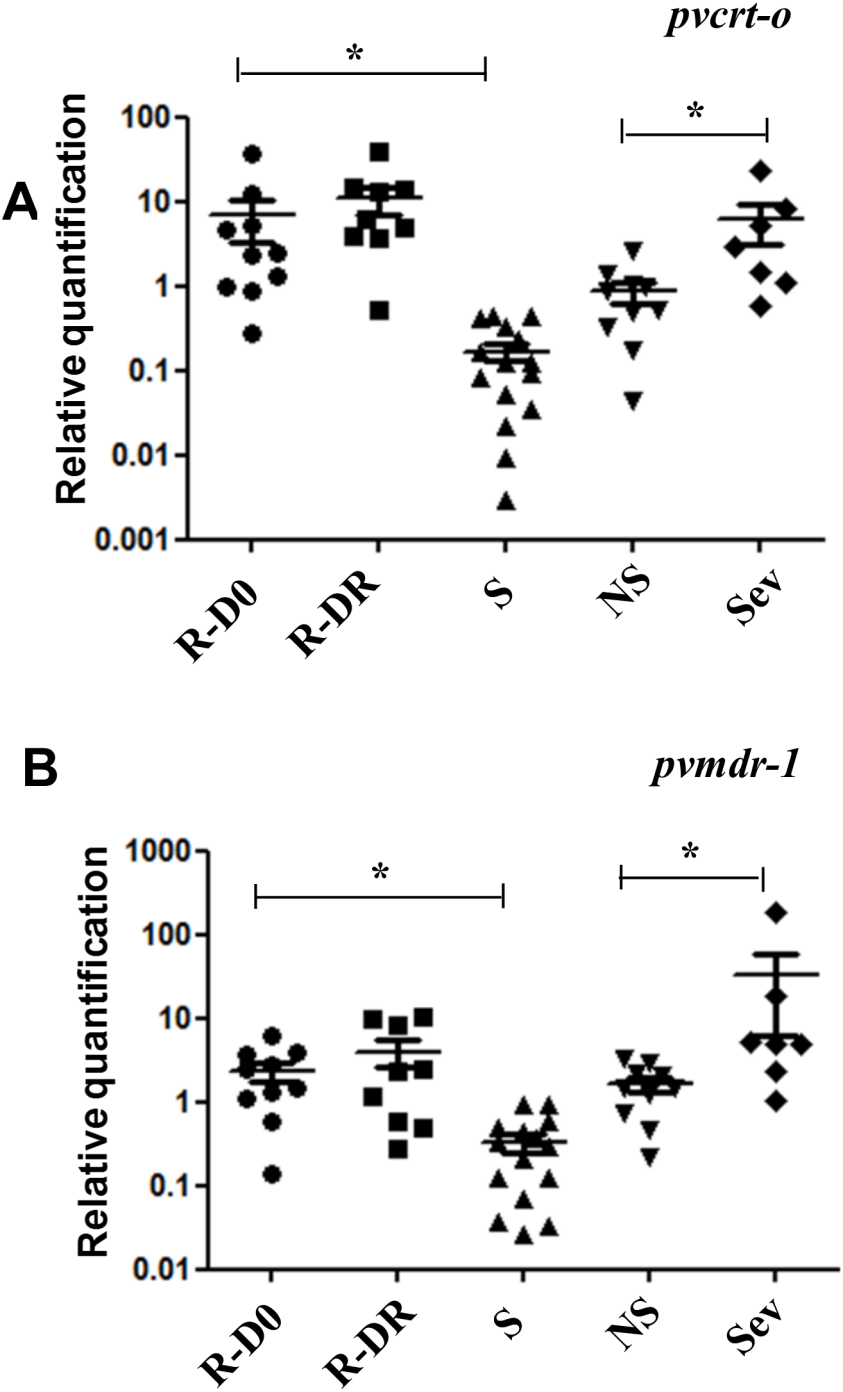
Expression gene levels of *pvcrt-o* and *pvmdr-1* in all the groups. Relative quantification of *pvcrt-o* (A) and *pvmdr1* (B) transcript levels in total RNA obtained from parasites from severe patients with chloroquine-resistant *P. vivax*, patients susceptible to CQ, severe patients and patients without severity symptoms. Chloroquine-resistant *P. vivax* parasites (R). Chloroquine-susceptible *P. vivax* parasites at D0 (S). Severe cases (Sev). Non-severe cases (NS). *p<0.05.

In patients R1 to R7 gene expression was analysed at D0 and DR, while in patients R8 to R10 and R11 to R12, due to insufficient RNA amount, gene expression was measured only at D0 or DR, respectively. Patients with CQ-resistant *P. vivax* parasites presented a higher gene expression of *pvcrt-o* and *pvmdr-1* at D0 and DR when compared to the susceptible group (Figure 1A and 1B). For the CQR patients, median gene expression values at D0 and DR, presented 2.4 fold (95% CI: 0.96–7.1) and 6.1 fold (95% CI: 3.8–14.3) increase in *pvcrt-o* levels (Figure 1A and 2A) compared to the susceptible patients at D0 with 0.12 fold (95% CI: 0.034–0.324) (Figure S1A and 2A). Median gene expression for *pvmdr-1* presented 2.0 fold (95% CI: 0.95–3.8) and 2.4 fold (95% CI: 0.53–9.1) increase levels at D0 and DR, for the CQR patients (Figure 1B and 2B) versus 0.288 fold (95% CI: 0.068–0.497) for the susceptible patients at D0 (Figure S1B and 2B). Patients R2, R4, R6 and R7 showed a higher expression level of both genes in relation to the control group at D0 and DR (Figure 1). It was not observed significant differences in expression levels between D0 and DR for resistant parasites (p = 0.375 for *pvcrt-o* and p = 0.844 for *pvmdr-1*). However, when we compared expression levels between resistant and sensible parasites at D0 for both genes, values were significantly greater for treatment failures (p<0.05 for both genes). No correlation was found between CQ concentration and gene expression of DR for *pvcrt-o* (p = 0.286) and *pvmdr-1* (p = 0.433) genes and between gene expression of *pvcrt-o* and *pvmdr-1* and concentration of RNA in D0 and DR (p>0.05).

To discard that difference in gene expression levels was not due to difference in the distribution of asexual blood stages, parasites distribution was counted in both, CQRPv and CQ-susceptible samples (Tables S4 and S5). Of notice, no significant differences were observed between immature and mature asexual forms in both groups (p>0.05). Moreover, it was found that the *pvcrt-o* and *pvmdr-1* gene mutations studied were not related to CQ-resistance (p>0.05) (Table 2).

**Table 2:** Frequency of polymorphisms in the *pvmdr1 and pvcrt-o* genes for the different set of patients.

Genotype	Number of samples (%)		P	Number of samples (%)		p
	Chloroquine-resistant	Chloroquine-susceptible		Severe malaria	Non-severe malaria	
***pvmdr-1***						
Mutant L1076 codon	0/12 (0.0)	0/15 (0.0)	NS	1/7 (14.3)	0/10 (0.0)	NS
Wild-type Y976 codon	12/12 (100.0)	15/15 (100.0)	NS	7/7 (100.0)	10/10 (100.0)	NS
***pvcrt-o***						
Wild-type without K10 insert	12/12 (100.0)	15/15 (100.0)	NS	7/7 (100.0)	10/10 (100.0)	NS

Not significant (NS).

In order to characterize the genetic variation for each individual population, two neutral microsatellite loci (*msp1*F3 and MS2) were analyzed in D0 and DR samples. The analysis of microsatellites showed at least one concordant allele (83.3%) in primary infection and recrudescence (Table 1). Patients which presented CQ concentration less than 100 ng/ml, showed the same microsatellite allele in D0 and DR. The expected heterozygosity (H_E_) was different in parasites from primary and recrudescence episodes (for *msp1*F3, primary: H_E_ = 0.915 and recrudescence: H_E_ = 0.681; and for MS2, primary: H_E_ = 0.531 and recrudescence H_E_ = 0.771).

### Gene expression levels in severe vivax malaria parasites

In the period of study, 70 patients were hospitalized with *P. vivax*. However, due to many comorbidities, and absence of any criterion of severity, only 16 could be characterized as severe vivax malaria. Of these, 7 patients presenting mono-infection by *P. vivax* with any criterion of severity were included. Of these patients, 4 had severe anemia, 1 had severe anemia paralleled with acute respiratory distress syndrome (ARDS) and acute renal failure, 1 had acute renal failure and prostration and 1 had ARDS alone. Demographic and clinical characteristics of the patients enrolled are presented in Table 3. Of these patients, 51.0% were male, with a mean age of 38.6 years. The mean disease duration was 6.3 days, median hemoglobin was 6.3 g/dl and mean parasitemia was 11,180 parasites/µl.

**Table 3:** Description of 7 patients presenting severe vivax malaria admitted to a tertiary health center, Manaus, Amazonas, Brazil.

Code	Sex/age	WHO severemalaria criterion	PCR	Microscopy	Duration offever (days)	Hemoglobin(g/dl)	Bilirubintotal (mg/dl)	Serumcreatinine(mg/dl)	Asexual malarialparasites/mm^3^
S1	M/43	Severe anemia	Positive	Positive	6	4.9	3.1	1.0	11160
S2	F/34	Severe anemia	Positive	Positive	1	4.8	8.1	0.9	34673
S3	F/41	ARDS	Positive	Positive	8	8.9	2.3	0.8	24814
S4	F/22	Severe anemia	Positive	Positive	8	6.4	0.4	0.6	264
S5	M/12	Prostation, acuterenal failure	Positive	Negative	6	5.4	0.9	7.2	0
S6	M/74	Severe anemia,ARDS, acuterenal failure	Positive	Positive	5	7.0	1.7	4.4	325
S7	M/44	Severe anemia	Positive	Positive	10	6.7	1.7	1.5	7000

Acute respiratory distress syndrome (ARDS).

**Figure 3:**
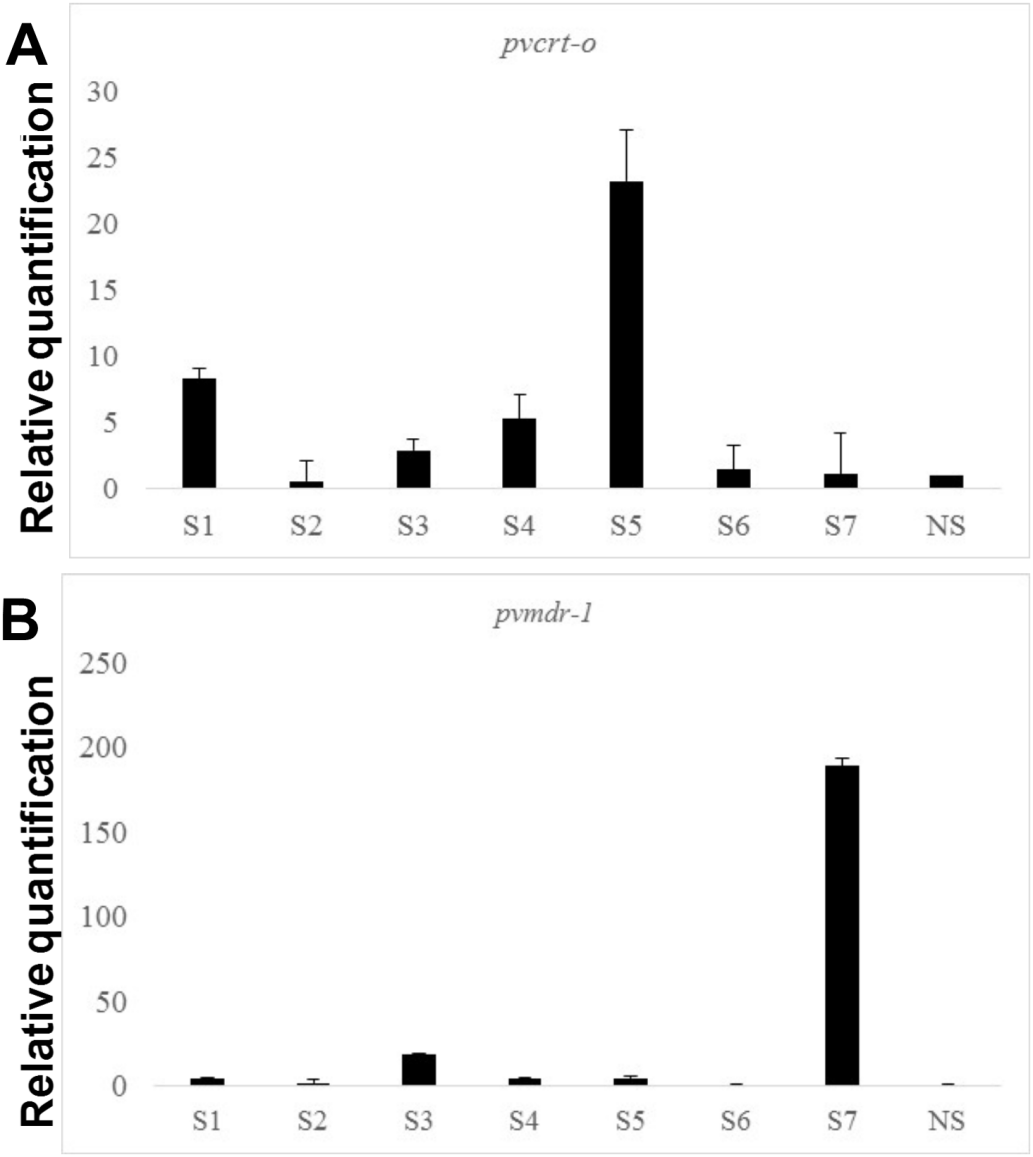
Expression level of chloroquine resistance genes in severe patients. Relative quantification of *pvcrt-o* (A) and *pvmdr1* (B) transcript levels in total RNA obtained from parasites from severe patients vs a pool of total RNA obtained from parasites susceptible to CQ. Severe cases (S). Non-severe cases (NS). The error bars in Figures 1 and 3 reflect the average standard error of the Ct.

Ten patients diagnosed with non-severe malaria were selected and included as controls. The same 5 CQ-susceptible isolates from patients with mild malaria above cited were used to generate the relative expression of seven severe malaria patients and 10 non-severe malaria patients. The group of patients with severe vivax malaria had a 2.9 (95% CI: 1.1–8.3) and 4.9 (95% CI: 2.3–18.8) fold increase in *pvcrt-o* and *pvmdr-1* expression level versus 0.7 (95% CI: 0.3–1.1) and 1.5 (95% CI: 0.7–2.4) for the control group with mild disease (p = 0.007 and p = 0.01) (Figure 2, Figure 3 and Figure S2). To discard that difference in gene expression levels was not due to difference in the distribution of asexual blood stages, parasites distribution was counted in both, severe vivax malaria (Table S6) and non-severe. No significant difference was found between immature and mature asexual forms among patients with severe and non-severe malaria (p>0.05) (Table S6). The *pvcrt-o* and *pvmdr1* gene mutations studied were not related to severity (p>0.05) (Table 2).

**Figure 4:**
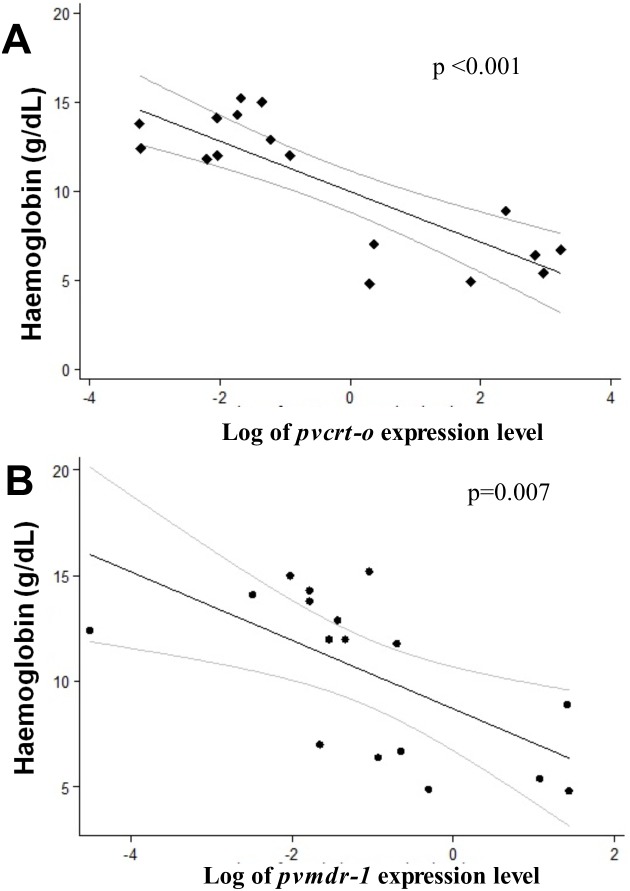
Correlation between expression gene levels *pvcrt-o* and *pvmdr-1* genes and hemoglobin concentration. Correlation between expression levels of chloroquine resistance genes, *pvcrt-o* (A) and *pvmdr-1* (B) and hemoglobin in hospitalized patient.

In a linear regression analysis, it was found a negative correlation between hemoglobin values (of severe and non-severe patients) and the log of the expression of the *pvcrt-o* (p<0.001) and *pvmdr-1* (p = 0.007) genes among hospitalized patients (Figure 4).

## Discussion

There have been recent reports on CQR from different regions of the world [8], [9], [15], [28]–[31], including Brazil [3], [10], [11]. Although the relevance of this problem, the molecular mechanisms of CQRPv are poorly understood. In this work, expression levels of *pvcrt-o* and *pvmdr-1* genes were determined in patients with CQRPv from a well-characterized cohort under supervised treatment, pointing to a link between *in* *vivo* CQR and overexpression of both genes. Furthermore, these patients showed higher concentrations of CQ and microsatellite revealed the presence of the same clonal nature at D0 and DR, bringing evidence that the re-emergent infection is the same as the primary infection, therefore indicating recrudescence.

It is likely that CQR contributes to the burden of severe vivax malaria in regions where resistance is emerging [4], [32], but there is paucity of available data regarding this issue. In this work, the group of patients with severe vivax malaria presented an increase in *pvcrt-o* and *pvmdr-1* expression levels, as compared to the control group with mild disease. These two genes have been suggested as genetic markers of CQR in *P. vivax* [33], [34].

Our results are in concordance with those reported by Fernandez-Becerra et al. [18], where high levels of *pvcrt-o* and *pvmdr-1* gene expression were observed in a patient with severe vivax malaria compared to three non-severe. A prospective study conducted in the Papua New Guinea highlighted *P. vivax* as a major cause of severe malaria, particularly in settings with established or emerging chloroquine resistance, supporting indirectly our findings [2]. One speculates that in areas in which CQR *P. vivax* circulates, acute febrile illness potentially becomes chronic, recurrent and severe, due to recurrent parasitemia [5]. Moreover, chronic infections could lead to diserythropoiesis, as is seen in *P. falciparum*-induced anemia [35].

In this investigation, CQR and severe *P. vivax* malaria were observed in parallel with a high expression of *pvcrt-o* and/or *pvmdr-1* genes in patients seen at a reference center in the Brazilian Amazon. Even though it is tempting to conclude about a causal association, a clear limitation of this study is that CQR has not been evaluated in the same patients with severe vivax malaria, especially because severe patients were in use of intravenous artesunate (ACT), making it impossible to evaluate CQ-susceptibility *in* *vivo* phenotype. Actually, patients with CQRPv were identified from following WHO criteria, excluding patients with any clinical complication [19]. Another clear limitation is the small number of samples used in this study. Moreover, so far it is not possible to rule out a differential virulence concurring with dissimilar drug resistance phenotypes. Studies on *P. falciparum* laboratory strains and field isolates of *P. vivax* suggest that there is marked variability in growth rates of isolates *ex* *vivo* with CQR isolates growing faster than CQ-susceptible isolates [36], [37]. Since parasites from patients with severe disease have greater *ex* *vivo* multiplication rates compared to those from patients with non-severe disease [38], the possibility arises that the highly CQR isolates may be more virulent.

Mefloquine (MQ) was used in the Brazilian Amazon until the beginning of the 2000s. Indeed, CQ and MQ resistance parallel in this region [8]. Thus, even after more than one decade of non-exposure to MQ, we could not discard this effect in the present. Actually, increased *pvmdr-1* copy number was found in two isolates in the Western Brazilian Amazon [39]. Unfortunately, *pvmdr-1* copy numbers were not measured in our samples.

In conclusion, our study suggests that CQR and severe *P. vivax* malaria are associated with increased expression levels of the *pvcrt-o* and *pvmdr-1* genes. CQRPv could be rising in the Brazilian Amazon and probably this fact could be contributing to the simultaneous spread of vivax malaria and clinical severity related to this parasite. Further studies are needed with larger number of patients and using other molecular markers to confirm the underlying pathogenesis of severe disease, and the degree to which this is related to the emergence of multidrug resistant strains of *P. vivax*.

## Supporting Information

Figure S1 **Expression levels of chloroquine resistance genes in patients susceptible to chloroquine treatment.** Relative quantification of *pvcrt-o* (A) and *pvmdr1* (B) transcripts in total RNA obtained from parasites from patients with chloroquine-resistant *P. vivax* vs a pool of total RNA obtained from parasites susceptible to CQ. Chloroquine-susceptible *P. vivax* parasites (S). Day admission (D0). Day of recrudescence (DR). The error bars reflect propagated error calculated with the average standard error of the Ct.
(TIF)

Figure S2 **Expression level of chloroquine resistance genes in non-severe** ***P. vivax*** **malaria.** Relative quantification of *pvcrt-o* (A) and *pvmdr1* (B) transcript levels in total RNA obtained from parasites from severe patients vs a pool of total RNA obtained from parasites susceptible to CQ. Non-severe cases (NS). The error bars reflect propagated error calculated with the average standard error of the Ct.
(TIF)

Table S1 **Oligonucleotide primers used for DNA sequencing of** ***P. vivax*** **orthologs genes.**
(DOC)

Table S2 **Oligonucleotides used for genotyping** ***P. vivax*** **parasites.**
(DOC)

Table S3 **Reproducibility and repeatability of measurements of Cts amplification of targets** ***pvcrt-o*** **and** ***pvmdr-1.***
(DOC)

Table S4 **Gene expression and different intra-erythrocytic stages of chloroquine resistance** ***P. vivax*** **parasites admitted to a tertiary health center, Manaus, Amazonas, Brazil.**
(DOC)

Table S5 **Different intra-erythrocytic stages of chloroquine-susceptible** ***P. vivax*** **parasites admitted to a tertiary health center, Manaus, Amazon, Brazil.**
(DOC)

Table S6 **Gene expression and different intra-erythrocytic stages of patients presenting severe vivax malaria admitted to a tertiary health center, Manaus, Amazonas, Brazil.**
(DOC)
