# Supplementary figures and images for: Expression Levels of pvcrt-o and pvmdr-1 Are Associated with Chloroquine Resistance and Severe Plasmodium vivax Malaria in Patients of the Brazilian Amazon
Source: PLoS One. 2014 Aug 26;9(8):e105922. doi: 10.1371/journal.pone.0105922 (PMC4144906; doi:10.1371/journal.pone.0105922)

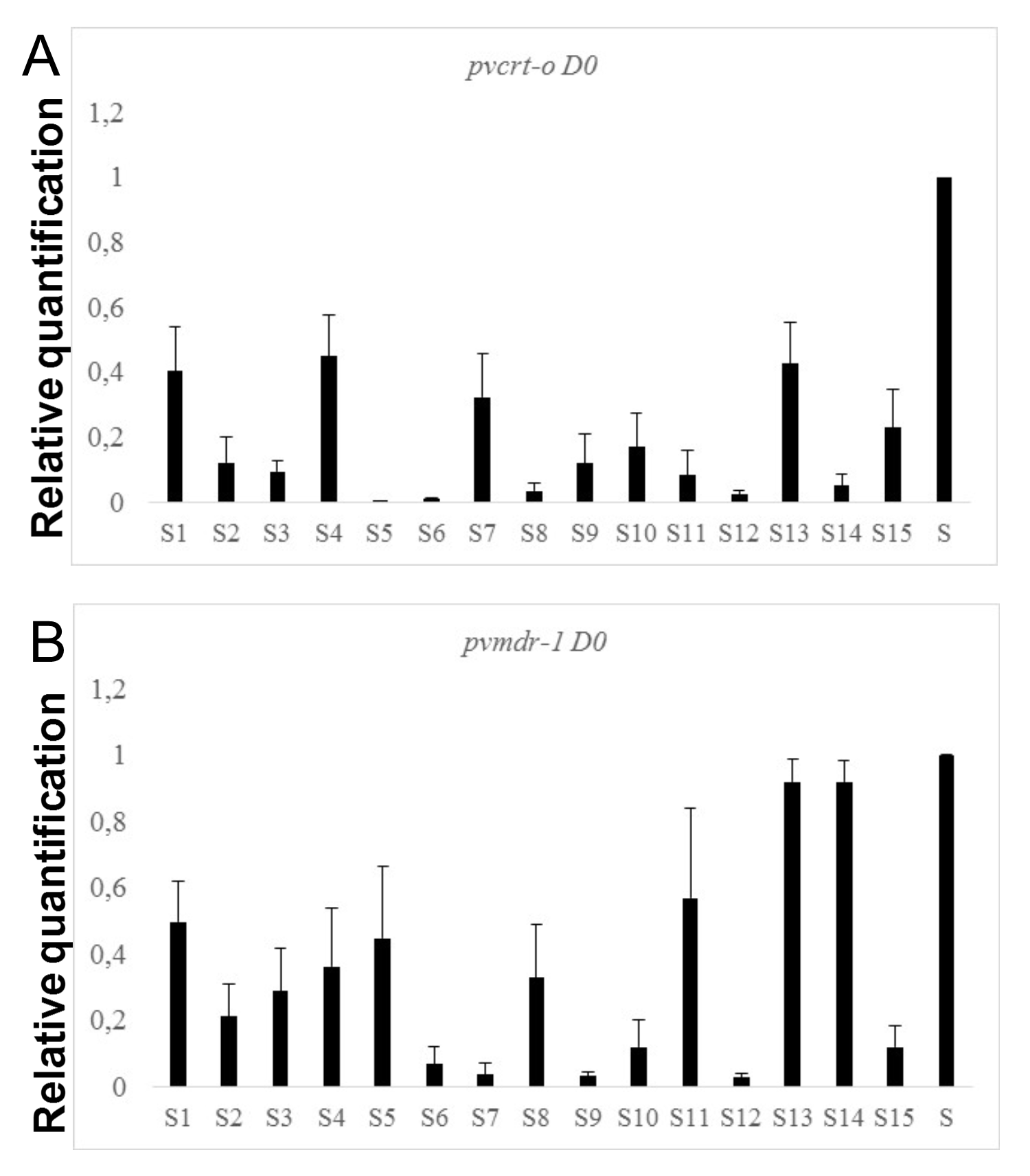

Supplement: Figure S1 — Expression levels of chloroquine resistance genes in patients susceptible to chloroquine treatment. Relative quantification of pvcrt-o (A) and pvmdr1 (B) transcripts in total RNA obtained from parasites from patients with chloroquine-resistant P. vivax vs a pool of total RNA obtained from parasites susceptible to CQ. Chloroquine-susceptible P. vivax parasites (S). Day admission (D0). Day of recrudescence (DR). The error bars reflect propagated error calculated with the average standard error of the Ct. (TIF) [file pone.0105922.s001.tif]

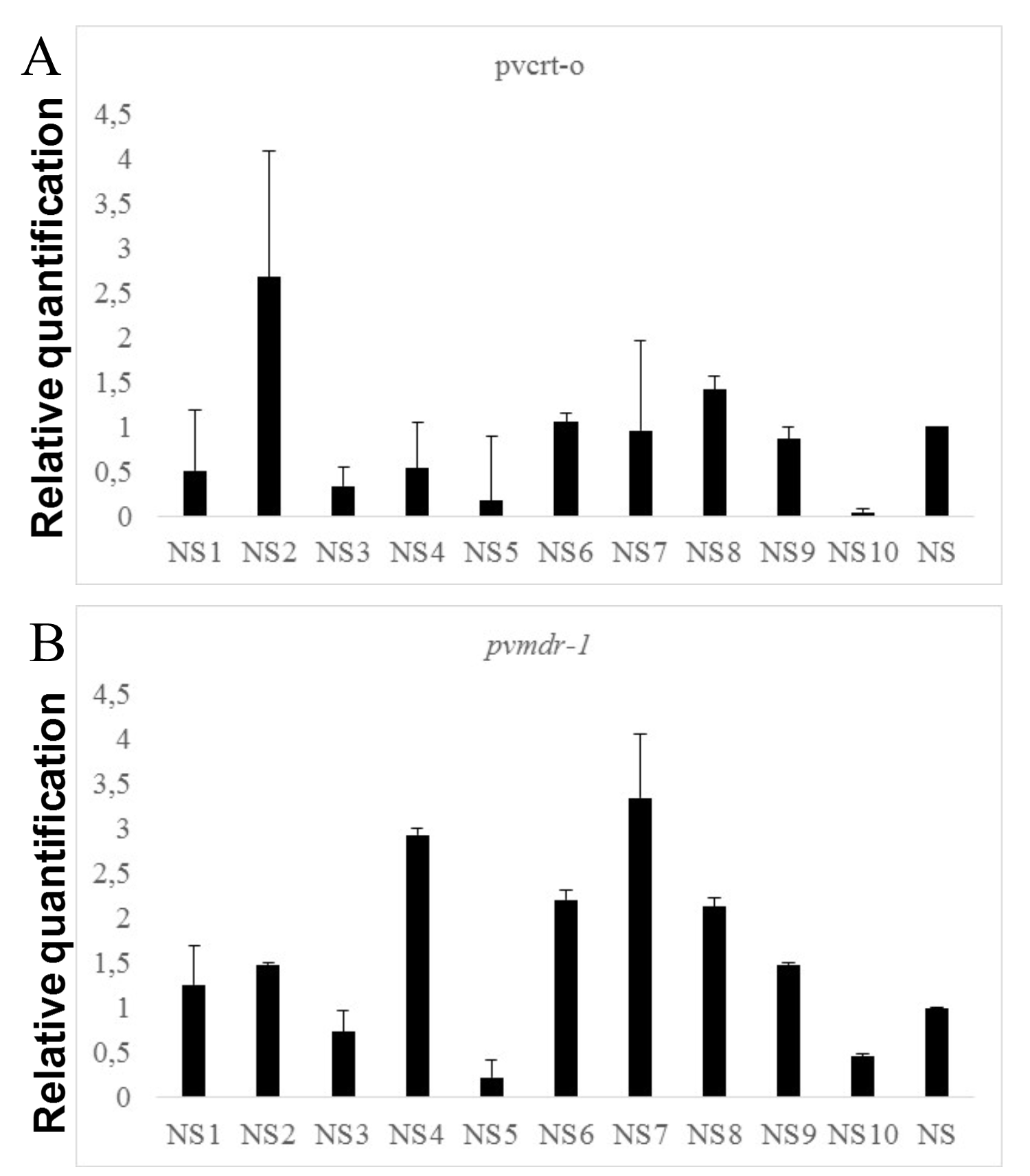

Supplement: Figure S2 — Expression level of chloroquine resistance genes in non-severe P. vivax malaria. Relative quantification of pvcrt-o (A) and pvmdr1 (B) transcript levels in total RNA obtained from parasites from severe patients vs a pool of total RNA obtained from parasites susceptible to CQ. Non-severe cases (NS). The error bars reflect propagated error calculated with the average standard error of the Ct. (TIF) [file pone.0105922.s002.tif]
