# Supplementary material for: Expression Levels of pvcrt-o and pvmdr-1 Are Associated with Chloroquine Resistance and Severe Plasmodium vivax Malaria in Patients of the Brazilian Amazon
Source: PLoS One. 2014 Aug 26;9(8):e105922. doi: 10.1371/journal.pone.0105922 (PMC4144906; doi:10.1371/journal.pone.0105922)
Supplement: Table S1 — Oligonucleotide primers used for DNA sequencing of P. vivax orthologs genes. (DOC) [file pone.0105922.s003.doc]

**Table S1. Oligonucleotide primers used for DNA sequencing of *P.vivax* orthologs genes.**

| **Gene** | **Chromosome** | **Sequence 5’ 🡪 3’** | **Base pairs** | **Use** | **References** |
| --- | --- | --- | --- | --- | --- |
| *pvcrt-o* | 1 | AAGAGCCGTCTAGCCATCC | 1,186 bps | Sequencing |  |
|  |  | AGTTTCCCTCTACACCCG |  |  |  |
| *pvmdr-1* | 10 | GGATAGTCATGCCCCAGGATTG | 604 bps | Sequencing |  |
|  |  | CATCAACTTCCCGGCGTAGC |  |  |  |

**References**

1. Lu F, Wang B, Cao J, Sattabongkot J, Zhou H, et al. (2012) Prevalence of drug resistance-associated gene mutations in plasmodium vivax in Central China. Korean J Parasitol 50: 379-384.

2. Imwong M, Pukrittakayamee S, Looareesuwan S, Pasvol G, Poirreiz J, et al. (2001) Association of genetic mutations in Plasmodium vivax dhfr with resistance to sulfadoxine-pyrimethamine: geographical and clinical correlates. Antimicrob Agents Chemother 45: 3122-3127.
