# Supplementary material for: Expression Levels of pvcrt-o and pvmdr-1 Are Associated with Chloroquine Resistance and Severe Plasmodium vivax Malaria in Patients of the Brazilian Amazon
Source: PLoS One. 2014 Aug 26;9(8):e105922. doi: 10.1371/journal.pone.0105922 (PMC4144906; doi:10.1371/journal.pone.0105922)
Supplement: Table S2 — Oligonucleotides used for genotyping P. vivax parasites. (DOC) [file pone.0105922.s004.doc]

**Table S2. Oligonucleotides used for genotyping *P. vivax* parasites*.***

| **Marker** | **Cromossome** | **Marker type** | **Primer name** | **Sequence 5' - 3'** | **Fluorescent dye** | **TM (ºC)** | **Mg** |
| --- | --- | --- | --- | --- | --- | --- | --- |
| MS2 | 3 | Microsatellite | primary forward | AGCACGACCAACAAGAGAGG | 6-FAM | 59 | 25 |
|  |  |  | nested forward | GAGCTAGCCAAAGGTTCAAA |  |  |  |
|  |  |  | Nested reverse | TGGGGAGAGACTCCCTTTTC |  |  |  |
| *msp1*F3 | 7 | Surface antigen | primary forward | GGAGAACATAAGCTACCTGTCC | VIC | 59 | 25 |
|  |  |  | primary reverse | GTTGTTACTTGGTCTTCCTCCC |  |  |  |
|  |  |  | nested forward | CAAGCCTACCAAGAATTGATCCCCAA |  |  |  |
|  |  |  | nested reverse | ATTACTTTGTCGTAGTCCTCGGCGTAGTCC |  |  |  |
