# Supplementary material for: Expression Levels of pvcrt-o and pvmdr-1 Are Associated with Chloroquine Resistance and Severe Plasmodium vivax Malaria in Patients of the Brazilian Amazon
Source: PLoS One. 2014 Aug 26;9(8):e105922. doi: 10.1371/journal.pone.0105922 (PMC4144906; doi:10.1371/journal.pone.0105922)
Supplement: Table S3 — Reproducibility and repeatability of measurements of Cts amplification of targets pvcrt-o and pvmdr-1. (DOC) [file pone.0105922.s005.doc]

**Table S3. Reproducibility and repeatability of measurements of Cts amplification of targets *pvcrt-o* and *pvmdr-1***

| **Target** | **First Run** | | | | | **Second Run** | | | | | **Mean Ct** | **SD** | **CV (%)** |
| --- | --- | --- | --- | --- | --- | --- | --- | --- | --- | --- | --- | --- | --- |
|  | **Mean Ct1** | **SD1** | | | **CV1 (%)** | **Mean Ct2** | **SD2** | | **CV2 (%)** | |  |  |  |
| *pvcrt-o* | 37,223 | | 0,501351 | 1,35 | | 36,4244 | | 1,010513 | | 2,77 | 36,8237 | 0,861804 | 2,34 |
| *pvmdr-1* | 35,1916 | | 0,261844 | 0,75 | | 35,696 | | 0,357134 | | 1 | 35,4438 | 0,397279 | 1,12 |

Threshold cycle value (Ct). Standard deviation (SD). Coefficient of variation (CV). Each value is the mean of 10 reactions.
