# Supplementary material for: Expression Levels of pvcrt-o and pvmdr-1 Are Associated with Chloroquine Resistance and Severe Plasmodium vivax Malaria in Patients of the Brazilian Amazon
Source: PLoS One. 2014 Aug 26;9(8):e105922. doi: 10.1371/journal.pone.0105922 (PMC4144906; doi:10.1371/journal.pone.0105922)
Supplement: Table S4 — Gene expression and different intra-erythrocytic stages of chloroquine resistance P. vivax parasites admitted to a tertiary health center, Manaus, Amazonas, Brazil. (DOC) [file pone.0105922.s006.doc]

**Table S4. Gene expression and different intra-erythrocytic stages of chloroquine resistance *P. vivax* parasites admitted to a tertiary health center, Manaus, Amazon, Brazil.**

| **Code** | ***D0*** | | | | | | | | | | | | **DR** | | | | | | | | | | | |
| --- | --- | --- | --- | --- | --- | --- | --- | --- | --- | --- | --- | --- | --- | --- | --- | --- | --- | --- | --- | --- | --- | --- | --- | --- |
|  | ***pvcrt-o* ge** | ***pvmdr-1* ge** | | **RNA conc** | | **%R** | | **%Trop** | | **%Schiz** | | ***pvcrt-o* ge** | | ***pvmdr-1* ge** | | **RNA conc** | | **%R** | | **% Trop** | | **%Sc** | |  |
| **R1** | 2.361 | | 0.136 | | 55.3 | | 86.7 | | 13.3 | | 0.0 | | 15.022 | | 0.272 | | 28.9 | | 90.0 | | 10.0 | | 0.0 | |
| **R2** | 5.261 | | 3.727 | | 83.6 | | 77.5 | | 20.6 | | 1.9 | | 3.687 | | 2.349 | | 50.7 | | 85.9 | | 9.6 | | 4.5 | |
| **R3** | 0.999 | | 1.339 | | 18.3 | | 90.0 | | 10.0 | | 0.0 | | 6.089 | | 10.011 | | 22.8 | | 88.5 | | 9.8 | | 1.7 | |
| **R4** | 37.554 | | 1.471 | | 18.0 | | 80.0 | | 15.5 | | 5.0 | | 38.377 | | 0.493 | | 114.1 | | 77.0 | | 23.0 | | 0.0 | |
| **R5** | 0.283 | | 0.580 | | 85.7 | | 80.0 | | 20.0 | | 0.0 | | 0.530 | | 0.58 | | 39.7 | | 71.4 | | 28.6 | | 0.0 | |
| **R6** | 4.570 | | 4.018 | | 16.2 | | 79.1 | | 16.7 | | 4.2 | | 3.863 | | 2.462 | | 39.8 | | 70.8 | | 25.0 | | 4.2 | |
| **R7** | 12.622 | | 1.084 | | 18.4 | | 60.0 | | 40.0 | | 0.0 | | 12.735 | | 1.165 | | 17.5 | | 71.4 | | 28.6 | | 0.0 | |
| **R8** | 2.482 | | 6.165 | | 16.5 | | 73.9 | | 24.9 | | 1.2 | |  | |  | |  | | 85.7 | | 13.0 | | 1.3 | |
| **R9** | 1.288 | | 2.490 | | 44.1 | | 58.8 | | 41.2 | | 0.0 | |  | |  | |  | | 78.9 | | 21.1 | | 0.0 | |
| **R10** | 0.851 | | 2.795 | | 58.9 | | 72.4 | | 27.6 | | 0.0 | |  | |  | |  | | 81.6 | | 18.4 | | 0.0 | |
| **R11** |  | |  | |  | | 33.3 | | 64.9 | | 1.8 | | 4.889 | | 8.139 | | 20.8 | | 81.8 | | 18.2 | | 0.0 | |
| **R12** |  | |  | |  | | 62.5 | | 37.5 | | 0.0 | | 13.504 | | 10.577 | | 89.4 | | 71.4 | | 28.6 | | 0.0 | |

Day admission (D0). Day of recrudescence (DR). Rings (R). Trophozoite (Trop). Schizont (Schiz). gene expression (ge). Concentration (Conc).
