# Supplementary material for: Expression Levels of pvcrt-o and pvmdr-1 Are Associated with Chloroquine Resistance and Severe Plasmodium vivax Malaria in Patients of the Brazilian Amazon
Source: PLoS One. 2014 Aug 26;9(8):e105922. doi: 10.1371/journal.pone.0105922 (PMC4144906; doi:10.1371/journal.pone.0105922)
Supplement: Table S5 — Different intra-erythrocytic stages of chloroquine-susceptible P. vivax parasites admitted to a tertiary health center, Manaus, Amazon, Brazil. (DOC) [file pone.0105922.s007.doc]

**Table S5. Different intra-erythrocytic stages of chloroquine-susceptible *P. vivax* parasites admitted to a tertiary health center, Manaus, Amazon, Brazil.**

|  | **S1** | **S2** | **S3** | **S4** | **S5** | ***S6*** | **S7** | **S8** | **S9** | **S10** | **S11** | **S12** | **S13** | **S14** | **S15** |
| --- | --- | --- | --- | --- | --- | --- | --- | --- | --- | --- | --- | --- | --- | --- | --- |
| **% Rings** | 76.2 | 80.0 | 83.3 | 44.4 | 40.0 | 82.4 | 29.4 | 38.9 | 77.5 | 54.6 | 66.3 | 61.4 | 38.9 | 87.7 | 73.3 |
| **% Trophozoites** | 23.8 | 16.0 | 16.7 | 55.6 | 54.6 | 17.6 | 70.6 | 61.1 | 20.0 | 36.9 | 33.7 | 36.9 | 61.1 | 12.3 | 26.7 |
| **% Schizonts** | 0 | 4.0 | 0.0 | 0.0 | 5.4 | 0.0 | 0.0 | 0.0 | 2.5 | 9.0 | 0.0 | 1.7 | 0.0 | 0.0 | 0.0 |

Chloroquine-susceptible *P. vivax* parasites (S).
