# Supplementary material for: Expression Levels of pvcrt-o and pvmdr-1 Are Associated with Chloroquine Resistance and Severe Plasmodium vivax Malaria in Patients of the Brazilian Amazon
Source: PLoS One. 2014 Aug 26;9(8):e105922. doi: 10.1371/journal.pone.0105922 (PMC4144906; doi:10.1371/journal.pone.0105922)
Supplement: Table S6 — Gene expression and different intra-erythrocytic stages of patients presenting severe vivax malaria admitted to a tertiary health center, Manaus, Amazonas, Brazil. (DOC) [file pone.0105922.s008.doc]

**Table S6. Gene expression and different intra-erythrocytic stages of patients presenting severe vivax malaria admitted to a tertiary health center, Manaus, Amazonas, Brazil.**

| **Code** | ***pvcrt-o* gene expression** | ***pvmdr-1* gene expression** | **RNA Concentration (ng/ml)** | **%Rings stages** | **%Trophozoite** | **%Schizont** |
| --- | --- | --- | --- | --- | --- | --- |
| **S1** | 4.890 | 8.334 | 40.6 | 77.8 | 11.1 | 11.1 |
| **S2** | 2.310 | 0.572 | 91.1 | 73.0 | 18.0 | 9.0 |
| **S3** | 18.840 | 2.906 | 102.4 | 54.1 | 41.7 | 4.2 |
| **S4** | 4.890 | 5.294 | 106.3 | 80.0 | 20.0 | 0.0 |
| **S5** | 5.147 | 23.256 | 230.2 | 79.6 | 20.4 | 0.0 |
| **S6** | 1.067 | 1.469 | 79.7 | 75.0 | 25.0 | 0.0 |
| **S7** | 189.112 | 1.091 | 20.1 | 74.6 | 25.4 | 0.0 |

|  |
| --- |
|  |
|  |
|  |
